# Categorizations of Trust and Distrust in the Classifications and Social Representations of Food among Pregnant and Breastfeeding Women in Spain—Applying the Cultural Domains’ Pile Sort Technique

**DOI:** 10.3390/ijerph20054195

**Published:** 2023-02-26

**Authors:** Araceli Muñoz, Cristina Larrea-Killinger, Andrés Fontalba-Navas, Miguel Company-Morales

**Affiliations:** 1 Training and Research Unit—School of Social Work, University of Barcelona, 08035 Barcelona, Spain; 2 Research and Innovation Group in Social Work (GRITS), TRU—School of Social Work, University of Barcelona, 08035 Barcelona, Spain; 3 “ToxicBody” Interdisciplinary Network, Department of Social Anthropology, University of Barcelona, 08001 Barcelona, Spain; 4 Research Group “Anthropology of Crisis and Contemporary Transformations (CRITS)”, Department of Social Anthropology, University of Barcelona, 08001 Barcelona, Spain; 5 CIBER de Epidemiología y Salud Pública (CIBERESP), 28029 Madrid, Spain; 6 Antequera Hospital, Northern Málaga Integrated Healthcare Area, 29200 Antequera, Spain; 7 Department of Public Health and Psychiatry, University of Málaga, 29016 Málaga, Spain; 8 Seron Primary Care Centre, Northern Almería Integrated Healthcare Area, 04600 Huercal-Overa, Spain; 9 Department of Nursing, Physiotherapy and Medicine, University of Almería, 04120 La Cañada, Spain

**Keywords:** cultural domains, pile sorts, food risk, trust, distrust, pregnancy, breastfeeding

## Abstract

Food is fundamental in the decision making of pregnant and breastfeeding women to care for their own health and that of their child. In this paper, we explore some common food classification systems and certain attributes assigned to these categories, represented by values of trust and distrust. This study is based on an interdisciplinary research project in which we analysed discourses and practices regarding the dietary intake of pregnant and breastfeeding women in relation to the presence of chemical substances in foods. The results presented are part of the second phase of this research where we explored the results of our analysis of the pile sort technique based on an analysis of cultural domains in order to explore the categories and semantic relations among terms regarding trust and distrust in food. This technique was applied to the 62 pregnant and breastfeeding women of Catalonia and Andalusia. These women also participated in eight focus groups that provided information and narratives enabling us to analyse the meanings of the associative subdomains obtained in the pile sorts. They classified different foods and assigned certain attributes to them according to the level of trust and mistrust, providing a social representation of food risks. The mothers expressed great concern about the quality of the food they consume and about its possible effects on their own health and on that of their child. They perceive that an adequate diet is one based on the consumption of fruits and vegetables, preferably fresh. Fish and meat generate serious concern, as their properties are considered ambivalent depending on the food’s origin and mode of production. These criteria are perceived by women as relevant to their food decisions and, therefore, emic knowledge should be taken into account when developing food safety programmes and planning actions aimed at pregnant and breastfeeding women.

## 1. Introduction

This article is based on an exploratory study of the food classification system and the values assigned to the resulting categories according to the criteria of trust and distrust by pregnant and breastfeeding women. Knowledge of how consumers perceive the differences between foods enriches our understanding of eating behaviours [1]. It is also important to be able to evaluate the risks present in food selection and consumption [2]. This includes both risks derived from habits and lifestyles [3] and those resulting from transformations in the food system for increased production, possibly involving manipulations that increase risks to health [4,5].

Cognitive models related to decision making in food choices include the attributes of trust–distrust which are, in turn, components of perceptions of food (in)security. Trust is an intangible construct with many definitions, ranging from the multidimensional encompassing principles such as competence, coherence and empathy tosociopsychological principles. The latter includes both the trust inspired by the institutional structures that regulate daily life and the calculating, rational and mediated by logic, assumptions and experience [6]. In any relationship, this trust is an essential component, albeit highly dynamic and fragile.

In such an important period of life as pregnancy and lactation, women need to feel sure that the food they eat is safe and accompanied by accurate information. This demand for assurance is all the stronger within the present global food system, which is complex and interconnected, and where it is difficult to trust the sources of food information [7]. The “nutritional cacophony” referred to by Fischler [8] is part of this globalised system and has an evident influence on food choices. Women’s nutrition, before and during pregnancy, plays a key role in their own health and in that of their child, and is an important aspect in optimising pregnancy outcomes [9]. For this reason, the concepts of confidence in food, related to its safety and perceived risks, are of cardinal importance in dietary preferences during pregnancy and breastfeeding [10,11].

Another important consideration is that the medicalisation of pregnancy [12,13] and nutrition [14] in Western countries, together with the biomedical discourses received, subject pregnant and breastfeeding women to incessant control and surveillance. Furthermore, in order to safeguard the health of their child (before and after birth), mothers are under great pressure to self-regulate and self-care [15,16]. Pregnancy and lactation, thus, are vital but stressful stages during which the woman may view what is happening to her body with fear and distrust [17], especially when subjected to the discourse of risk. As a result, many women apply precaution as a strategy for managing uncertainty [18] and for protecting the child [19]. As a consequence, food-related discourses and practices are a vital consideration in women’s attitudes towards their health.

In this fundamental place that food occupies in the decision making of pregnant and breastfeeding women for the care of their own health and the baby’s [20,21], the criteria applied for food choice and consumption depend on various socioeconomic and cultural factors [18,22].

In this paper, we analyse food classification systems and certain attributes assigned to these categories, represented by values of trust and distrust. An analysis of cultural domains [23], an area of cognitive anthropology, brings us closer to how members of a society think about certain sets of items that have a joint presence in their culture or which are represented as being of the same type [24,25]. Analyses of cultural domains are commonly used in medical anthropology, and there exists an extensive bibliography regarding this type of research. In the field of food and nutrition, the pile sort technique is especially useful [26,27,28,29,30,31,32,33,34].

## 2. Materials and Methods

### 2.1. Study Design and Setting

This study, which was part of a broader interdisciplinary research project, was conducted to analyse discourses and practices on the dietary intake of pregnant and breastfeeding women in relation to the presence of chemical substances in foods [11,18,22,35,36,37]. The research was conducted in two phases, the first from 2015 to 2017 (Ref. CSO2014-58144-P) and the second from 2018 to 2021 (Ref. AP-0139-2017).

The field work was carried out at various health centres (hospitals and primary care centres) in the Spanish autonomous regions of Catalonia (Barcelona and its metropolitan area, Baix Llobregat, Tarragona and Ribera d’Ebre) and Andalusia (Granada and its surroundings, Valle del Almanzora, Antequera and Cabra).

### 2.2. Study Sample

The sample selection process was intentional or purposive, seeking the maximum variation, heterogeneity and intensity, whilst obtaining a balanced sample with similar representations of age, education, occupation and socioeconomic stratum. The following inclusion criteria were applied: women born in Spain, 20 weeks pregnant or more, or who had given birth during the last six months and were breastfeeding (exclusively or also using formula). The exclusion criteria were that the women must not have had any pathology that entailed a change in their diet. All participants were informed of the objectives and methods of the research and gave written consent to take part. Approval was also obtained from the corresponding ethics committees.

### 2.3. Data Collection and Analysis

**Figure 1:**
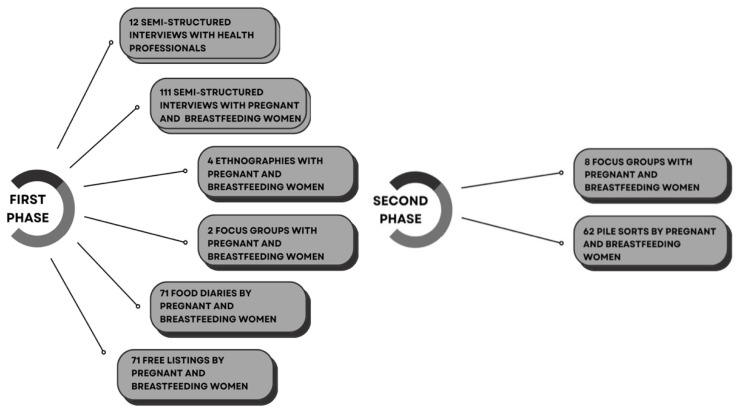
First and second phases. Data collection instruments.

In the first phase of the research (Figure 1), 111 semistructured interviews were conducted (with 62 pregnant women and 49 breastfeeding women) together with 4 focused ethnographies, 2 focus groups, 71 food diaries, 71 free listings and 12 interviews with health professionals (Table 1). In the second phase, eight focus groups and 62 pile sorts were conducted with 62 mothers (26 of whom were pregnant and 36 of whom were breastfeeding) (Table 2).

In Muñoz et al. [36], we reported the results of our analysis of the data obtained from the free listings carried out in the first phase of the study, in which the mothers were urged to think about what types of food they saw as trustworthy and untrustworthy and to make lists of each type of food. In the article, we explained how the technique of free listings was applied to a group of pregnant and breastfeeding women to analyse the main shared items or elements regarding trust and distrust in food. This technique is based on an analysis of cultural domains [23], a method for analysing social meanings and shared knowledge [24], which enables us to understand how mothers assimilate different social meanings and to determine the most important categories used in talking about trust/distrust in relation to food.

In this paper, we present the second phase of the study, in which we conducted a different technique that was also based on the analysis of cultural domains [23] in order to analyse categories and semantic relations among terms regarding trust and distrust in food [25]. This technique, pile sort, was applied to the 62 pregnant and breastfeeding women who participated in the eight focus groups. In this approach, free lists are usually followed by pile sorts [25] to identify the relations among terms within a given domain [38]. The basic aim of this technique is to formalise an associative cultural map for each of the indicated areas. Pile sorts are mainly used to obtain semblances between certain items in a cultural domain or attributes that are used to distinguish these items from the informants’ criteria [39].

#### 2.3.1. Items or Categories of Analysis from the Free Listings

The participants were instructed to group the main items/categories of food cited in the free listings obtained in phase one of the study, reflecting similarities or differences regarding their trust and distrust in each case. Thus, the food categories for trust (*n* = 20) and distrust (*n* = 20) used in the pile sorts were obtained from the free listings, i.e., the twenty most cited items in the free listings for trust and distrust, respectively (Table 3).

The mothers were asked to identify different types of food as trusted or distrusted and to attribute qualities and adjectives to each product together with the specific properties perceived and other relevant characteristics related to the origin, manipulation, processing and distribution of the product. Adjectives such as fresh, natural, organic, whole-grain, seasonal, local, from the garden, homemade, craft or washed are often associated with trust, while those termed as processed, industrial, precooked, prepared, packaged, canned, fried or foreign tend to be distrusted. It is important to note that some foods are mentioned several times, since distinctions are made depending on their handling.

#### 2.3.2. Item Sorts and Categories of Analysis

The pile sorts were obtained as follows: each participant was given two sets of cards, one with the names of types of food that generally inspire trust and the other with those associated with distrust [38]. Each set of cards was randomly shuffled, and the participants were instructed to group the cards by sorting them into piles with as many or as few cards as they wished. No specific criteria for doing so were mentioned [40]. However, the women were told they could not put all the cards in a single pile [38].

#### 2.3.3. Analysing with ANTHROPAC and Nonmetric Multidimensional Scaling (nMDS)

The information from the pile sorts was classified and analysed using *ANTHROPAC* software (version 1.0.1.36, Software for Cultural Domain Analysis, Borgatti, SP.; Analytic Technologies: Natick, MA, USA, 2003), designed for the quantitative analysis of qualitative data and cultural domains [41]. From the *ANTHOPAC* findings, *nonmetric multidimensional scaling (nMDS)* was performed on the participants’ trust and distrust in the food categories, to represent the pile sorts obtained that reflected the proximity/distance of each category in the mother’s perceptive universe. *nMDS* provides a way to represent semantic proximities without requiring metric data [42], in which the distances between items reveal correlational, not metric distances [25]. The outcome of this process is a graphic display of the mothers’ thought processes in creating the pile sorts [43].

#### 2.3.4. Identifying Clusters or Dimensions

*nMDS* results may be interpreted by considering the items as dimensions or as clusters [25]. In our analysis, the associative subdomains in the women’s perceptions were overlaid on the representation. The *nMDS* model enables various interpretations to be made, and so the item areas or dimensions proposed are tentative. Using the information obtained from the focus groups, we identified clusters or dimensions and decided which labels should be attached to each [29]. In the case in question, five clusters or dimensions in the *nMDS* were related to trust and another five to distrust (Table 4). The labels of these dimensions refer not only to different types of foods but also to the qualities and properties attributed to them, as well as other characteristics related to their origin, manipulation, processing and distribution.

#### 2.3.5. Pile Sorts and Focus Groups’ Narratives

The focus groups provided information and narratives enabling us to analyse the meanings of the associative subdomains obtained when the mothers constructed pile sorts. The focus groups also informed us about their sociocultural norms, attitudes and perceptions regarding interactions with the environment [44]. The participation of these women in the focus groups gave us a better understanding of the social contexts in which crucial decisions were made. These qualitative data were analysed following the strategies expressed in Grounded Theory [45,46] to identify, interpret and explain the core meaning of the data obtained from pregnant and breastfeeding women and thus to generate meaningful codes and categories. The study information was exhaustively systematised using *ATLAS-ti* qualitative analysis software (Version 8; ATLAS-ti Scientific Software Development GmbH: Berlin, Germany, 2019).

## 3. Results

We present the results obtained from analysing the pile sorting activity and the focus groups’ contributions regarding associative subdomains for trust or distrust in different types of food. This analysis enabled us to distinguish similarities and differences in the categories generated by the focus group participants.

In these focus groups, the participants remarked on the complexity of determining which foods can be trusted and which cannot. In relation to food environments, for example, they reflect on how contamination may affect production and provoke distrust in the food consumed:

“Absolutely. For me, this has a direct influence, because the plants, which rely on rain, on the water that falls, when they’re in contaminated ground, this is where they feed. And the animals that eat contaminated grass, or that eat, well, everything… the water, everything, the nitrates, everything that’s in the soil, then everything ends up in the plant. And we eat all of that. So, yes, I do think it affects us”.

*(Tarragona focus group)*

They also note that distrust in some foods spurs a search for more information, which can then provoke even greater distrust in the products investigated:

“Of course, you tend to distrust everything. Then again, if you have to look at what’s in all the food, you wouldn’t buy anything, you wouldn’t eat anything. But finally, you end up eating it because you like it, full stop…”.

*(Tarragona focus group)*

The pregnant and breastfeeding mothers consulted expressed great concern about food quality and its potential effects on their health and that of their child, and emphasised the importance of knowing what they are eating, on reading the labels and on having good information about the composition of the products bought and consumed:

“You try to take better care of yourself. Also, now that I’ve got over the gestational diabetes I had, well, I look at everything much more closely and now I’m looking at the labels much more than I used to, I can see they add sugar to things, which I didn’t know about before, I didn’t… For example. So, it’s true that now, apart from being careful with freezing and washing food, and I don’t know what else, when you start looking at the labels that’s another question... In my case, due to my special circumstance, yes. When this is all over, sure, I won’t look at them so carefully”.

*(Antequera focus group)*

“Take fish, for example. Even if it’s fresh today, I don’t care. Even if it’s fresh today, I won’t eat it straight away. I always freeze it and eat it later, because once before, I was going to eat it on the same day and I found this little worm inside, the larva or whatever, though later I heard that you can’t see anisakis, it’s impossible. Well, I saw a worm, I don’t know if it was anisakis or what it was, but from then on, I’ve always frozen everything”.

*(Tarragona focus group)*

The women participating in the focus groups also argued that distrust is not incompatible with taste, since many of these foods are liked; nevertheless, they are avoided because they are not considered healthy. Distrust in itself does not prevent consumption. Some of the women acknowledged that you should not think too much about food because otherwise you would end up not eating anything. As some of the participants pointed out: “Everything creates distrust, but distrust doesn’t stop you from eating” *(Tarragona focus group)*, or “We don’t trust it, but we have to eat” *(Tíjola focus group).*

Another idea that came out in these narratives, and one that makes deciding which foods to choose even more complex, is that the amount of food or the frequency of its consumption is important. In other words, the excessive or abusive consumption of any product can have a negative impact on people’s health.

“- …. But if you haven’t got much time, and now you’re going to do the shopping, are you going to look at the label on every single product? I don’t know, that would drive you … you’d spend all day in the supermarket.

- What I do, at most, is to look later at home, though… the first few times I’ve bought something…

- I always buy the same things, that’s all. But at first, I used to stop and pay good attention to the E, the stabilisers, the sweeteners, the acidulants, the E340, the E three hundred and whatever. Because I remembered really well that here in Cabra, at school, the teacher sent us all to the supermarkets to write down all the E’s in the food products. So, later on, we were well aware of what we were doing … dairy this, meat that, whatever the other, and we realised that almost everything was carcinogenic.

- If you start reading, you won’t eat anything”.

*(Cabra focus group)*

### 3.1. The Trust of Pregnant and Breastfeeding Women in Food

**Figure 2:**
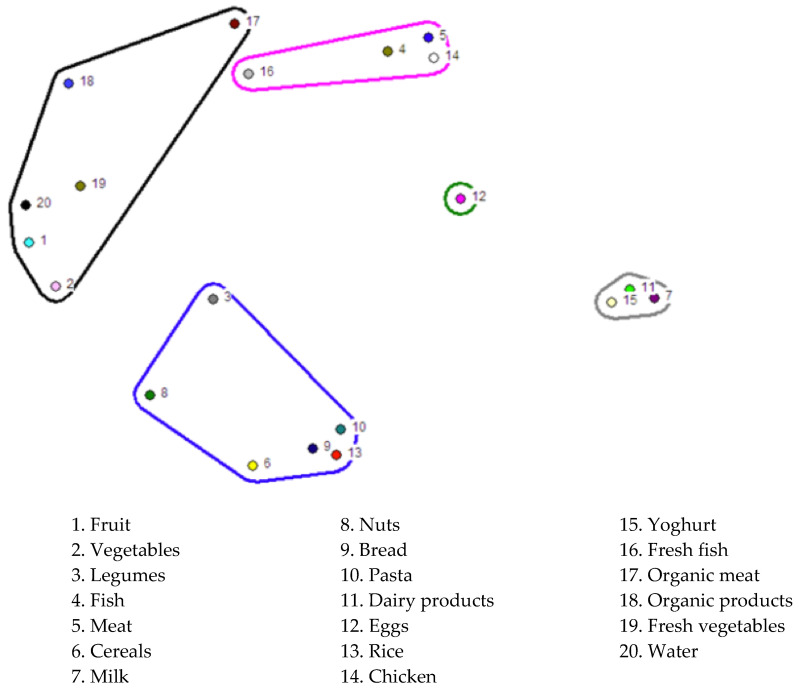
*Nonmetric multidimensional scaling (nMDS)* of trust in food. Representation of the total number of pile sorts (*n* = 62), showing the proximity/distance of the categories in the women’s perceptual universe. The *nMDS* model allows for various interpretations, and therefore the proposed item areas are tentative. Interpretation of the clusters or dimensions: blue: cereals, legumes and nuts; black: trusted items due to the nutritional properties of the food itself and/or its origin and manipulation; pink: items characterised by ambivalence regarding (mis)trust; green: eggs; grey: dairy products.

The *nonmetric multidimensional scaling (nMDS)* performed for confidence in food revealed five associative subdomains in the proximity/distance map of the categories, according to the perceptions of the women participating (Figure 2).

The clusters or associative subdomains of the *nMDS* graphical representation of trust in food were given these labels: 1. items in which trust arises from the nutritional properties of the food itself and/or its origin and manipulation; 2. cereals, legumes and nuts; 3. dairy products; 4. eggs; 5. items in which participants expressed ambivalence in terms of (mis)trust.

**Figure 3:**
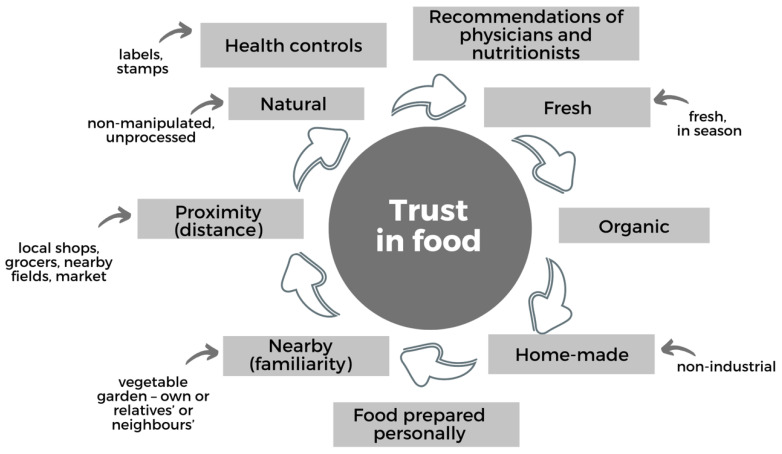
Categories and items arising from the focus groups in relation to trust in food.

In the focus groups, various categories and items related to trust in food emerged from the participants’ narratives (Figure 3).

The first area of items in the *nMDS* model of trust in food revealed two related categories: on the one hand, fruits and vegetables, in which trust is based on their inherent nutritional properties; on the other, organic meats and products linked by trust based on their origin and handling.

“In relating these groupings with the narratives drawn from the focus groups, it can be seen that, in reference to trust in the nutritional properties of fruits and vegetables, the participants remarked that natural, fresh products contain the best nutritional components, vitamins and minerals. Furthermore, these nutrition-based attitudes towards the properties of foods are reinforced by biomedical care during pregnancy and childbirth, although taste also plays an important role; thus, some mothers emphasised that fresh fruit had a better flavour: ‘Exactly, so they can grow them faster, because when you see a tomato that’s this big, but when you eat it, it doesn’t have any taste!’”.

*(Tíjola focus group)*

Although fruits and vegetables are trusted and considered to be healthy and nutritious, some of the women consulted compared the use of pesticides and fertilisers in intensive agriculture (unfavourably) with the absence of these products from their own and relatives’ vegetable gardens. They provided an environmental justification in relation to a growing distrust toward chemical substances derived from agricultural production [18,36], and they argued that intensive agriculture harms plant health and growth by degrading the soil and water. Some of the women commented that although they tried to avoid using pesticides in their gardens, this was not always possible:

“Because they’ve made [the fruit, vegetables] bigger, oh yes, with fertilisers and the like... I know that my father-in-law has to use something to kill the bugs, because nowadays the soil isn’t good, the water isn’t good. So, I don’t know, your plants die. If you don’t use something extra, the tomatoes won’t grow”.

*(Tíjola focus group)*

Nevertheless, these women distinguish between the products of intensive agriculture and what comes from their own or their family’s gardens, which they know is much healthier:

“My father-in-law has a vegetable garden and we used to help him out. The best thing is... there’s no comparison, you pick a tomato from there... the bunch of tomatoes or the peppers in season, … we grew it ourselves, that’s what I really trust”.

*(Cabra focus group)*

In relation to organic meat and other organic products, and people’s trust based on the origin and handling of the food, the participants in our focus groups remarked that they had more trust in this type of product, which they considered less industrially manipulated and contained fewer chemical additives such as pesticides, herbicides and hormones. In their opinion, organic certification gives a sense of security backed by production controls, where the legal discourse of certification generates trust associated with healthy living and sustainability. A woman who works in a meat producing company shared her experience:

“- Even if there are lots of controls, industrial methods will never be as natural as what you do at home. But it is true that the industrial scene has all the veterinary controls. When you slaughter at home, well yes, the vet is usually there. Except when he isn’t. That’s the thing. So, it’s a bit complicated.

- And when you buy from the local man, you don’t know if the pig has been sick, either...

- That’s right.

- And, for example, the animal could have been given antibiotics, right?

- Sure. So, I know that my company has passed all the quality controls. I would trust the food completely if I did this at home, you know, but someone else who does it, well, you don’t know what they’re feeding the pig, you don’t know if the vet has been to... to make sure that the meat is in good condition. So, really, I’d eat my company’s meat, that’s the truth”.

*(Tíjola focus group)*

The *nMDS* model of consumer trust also contained a second set of items that were a little more widely separated: an associative subdomain comprised of cereals, legumes and nuts.

In their words, the members of the focus groups trusted cereals, viewing them as products that usually keep well without spoiling. In this respect, they placed special emphasis on bread, pasta and rice, which were termed “balanced” foods and a source of energy.

“What would give me the most confidence would be, as I said before, pasta and legumes, because they give me more peace of mind”.

*(Antequera focus group)*

The levels of trust in cereals were determined by notions of their origin, price and degree of manipulation.

“For example, because I see it in my work, maybe... “Look, I’m going to buy these cereals for my child, because they’re the best, because they are more expensive”, and you compare them with another brand and it has a lot of added sugar…”.

*(Cabra focus group)*

Many of the participants trusted legumes because this type of food has many nutritional properties with proteins and iron. For these mothers, it is important that legumes are subject to very little processing and manipulation and are easy to store.

“Well, the thing is to have a balanced diet, a little of everything. With legumes, fish, a bit of everything. In pregnancy, sometimes, I don’t know, sometimes it does something to you, but well. Sure, dairy products and all that, yes”.

*(Tarragona focus group)*

It is these very characteristics that are apparent in legumes, which are seen as trusted products, like nuts. The mothers in the focus groups also indicated that legumes are very “natural”, being rich in healthy fats and providing a lot of energy.

“For I eat a few nuts, too. They give me energy, I think. I don’t know if it’s psychological, but I think they do me good”.

*(Sant Feliu focus group)*

“Nuts, perhaps, is what I trust most. Because I think everything else is more adulterated, and … With animals, it’s hormones. With fish, heavy metals. Fruit and vegetables, they’re full of pesticides. Unless you’re going to buy organic, then you know there are more guarantees. Nuts, I would say, maybe. And seeds”.

*(Tarragona focus group)*

Considered in a similarly positive light are two associative subdomains, dairy products and eggs.

Dairy foods, especially milk and yogurt, also generated confidence among the participants, who referred to these products as complete and nutritious with a high calcium content and which undergo strict controls in their preparation process.

Eggs were also among the foods that were generally well trusted by the mothers in the study groups, who emphasised that it provides lots of protein and is subject to little industrial manipulation. However, this trust depends on the production methods used; eggs from free-range hens are trusted because this status affects the way the hens are treated, their behaviour and, ultimately, the quality of the food. By contrast, the intensive production model is criticised, and this negative view influences consumer choice.

“Eggs, for example, before, the chickens lived free, they were... now we eat eggs from chickens that are stressed out because they are locked up. It’s true, they are psychologically stressed, they’re pressured to lay eggs, come on, lay eggs that need to be sold, do this, do that… I don’t like eggs very much. I’ll often buy from someone who’s got a bit of land, I’ll buy just a few eggs from them... I also buy in supermarkets, because you’ve got to do the shopping, ...”.

*(Tíjola focus group)*

Finally, another set of items in the *nMDS* trust model formed an associative subdomain characterised by ambivalence between trust and distrust. This ambivalence concerned two types of food in particular: fish and meat.

On the one hand, fish generated trust due to the properties of the food itself, its proteins, minerals and omega 3 content. On the other hand, distrust could occur depending on the origin, manipulation and conservation of this food. As one of the mothers pointed out:

“For example, I eat a lot of fresh fish, my father goes out fishing, and you can’t even trust that, really, because of the plastics in the water, the spills from the boats...”.

*(Vera focus group)*

Meat is another food that generated trust due to its natural properties, providing proteins and containing a lot of iron. However, concerns about production and manipulation reduced the level of trust in this respect as well.

“- I prefer to go to the butcher’s and have the meat minced there, rather than buy packaged minced meat from...

- But you don’t know what you’re eating from the butcher’s either. I can tell you, because I work a lot there.

- Yes, of course, obviously... But at least you can see the piece of chicken and the piece that they’re mincing.

- They put it in the display case and say, how good it looks, how clean it is, but they also add salt water and leave it for 24 h to turn white...

- Yes, of course, of course.

- At present, I’d rather trust the meat, for example, from the M. supermarket than from the butcher’s. In my experience, anyway”.

*(Antequera focus group)*

“Well, the thing is to have a balanced diet, a little of everything. With legumes, fish, a bit of everything. In pregnancy, sometimes, I don’t know, sometimes it does something to you, but well. Sure, dairy products and all that, yes”.

*(Tarragona focus group)*

### 3.2. The Distrust of Pregnant and Breastfeeding Women in Food

**Figure 4:**
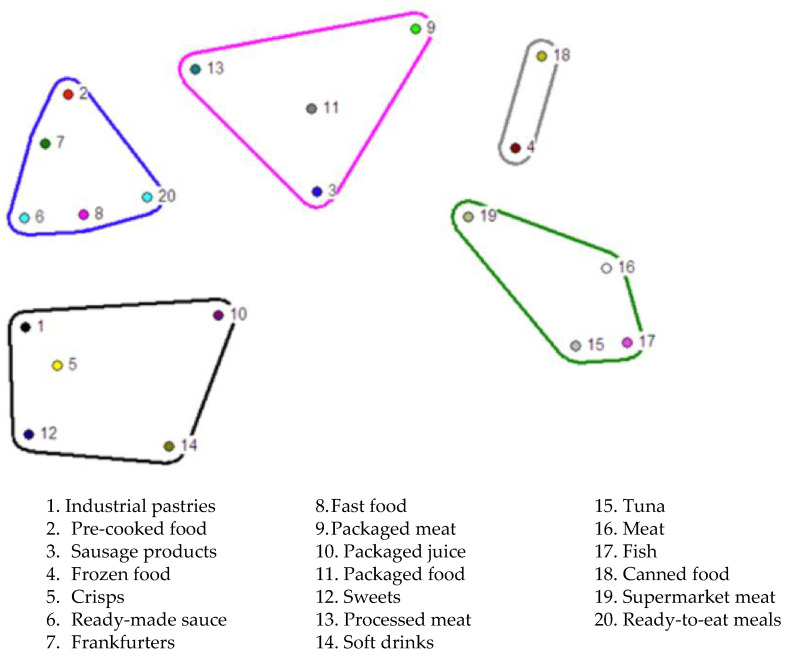
*Nonmetric multidimensional scaling (nMDS)* of distrust in food. Representation of the total number of pile sorts (*n* = 62) showing the proximity/distance of the categories in the women’s perceptual universe. The *nMDS* model allows for various interpretations and therefore the proposed item areas are tentative. Interpretation of the clusters or dimensions: blue: prepared or pre-cooked food; black: highly artificial processed products with many added chemical substances; pink: basic processed products; green: products that are distrusted due to their origin, manipulation and/or distribution; grey: products that are distrusted because of concerns about added preservatives.

The illustration of *nonmetric multidimensional scaling (nMDS)* on distrust in food shows that there are five associative subdomains in the proximity/distance map of the food categories according to the pregnant and breastfeeding women participating in this study (Figure 4).

The different clusters or associative subdomains in the *nMDS* graphical representation of distrust in food are labelled as: 1. prepared or precooked products; 2. basic processed products; 3. highly artificial processed products with many added chemical substances; 4. items distrusted due to their origin, manipulation and/or distribution; 5. items distrusted due to concerns about added preservatives.

**Figure 5:**
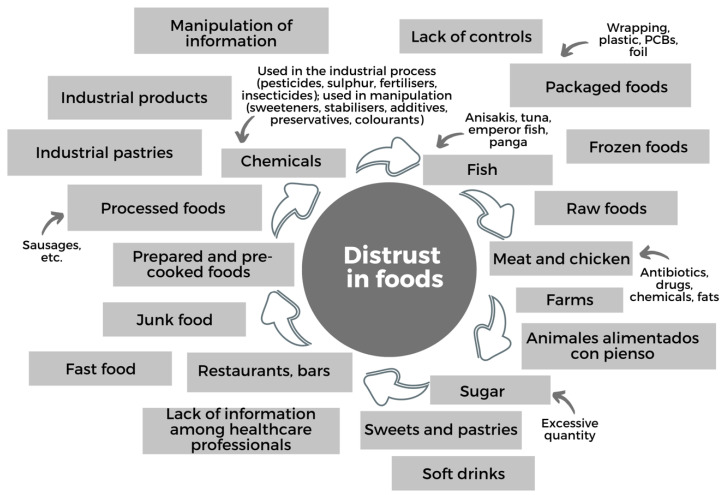
Categories and items arising from the focus groups in relation to distrust in food.

The discussions held in the focus groups gave rise to the following categories and items related to trust in food (Figure 5).

Continuing with the first area of items in the *nMDS* model of food distrust, there were three subdomains of items that grouped a large body of foods that generated suspicion according to the way in which they were manipulated: these were prepared or precooked products, basic processed products and highly artificial products with chemical additives.

In relating these groupings with the narratives from the focus groups, it can be seen that the participants distrusted prepared or precooked products because they do not know what substances they may contain; they also had serious reservations concerning how these foods are processed, manipulated, cooked, preserved and packaged.

“- Very good. What foods do you think contain chemicals?

- Almost everything.

- It depends. If you are going to buy fresh, well…

- Pre-cooked ones, definitely.

- Fresh ones, too, now... there’ll be something in them.

- The fresh ones will have less. Fresh products always have… there’ll be less, that’s why they have a shelf life. But there’ll be preservatives in canned goods, preserves, all of that”.

*(Vera focus group)*

In relation to basic processed foods, such as meat, the participants said they distrusted those that have been “processed” or “treated”, referring in particular to the addition of preservatives, stabilisers and colourants, which they perceived as “unnatural”.

“- Yes, I’m not so keen on packaged food. For example, take minced meat; if you read the label on the pack you’ll see it contains more things that are not meat than what is meat. But if you go to the butcher’s, they’ll be mincing it right there, in front of you, to give an example. Or pork loin, when you see it’s full of water or, I don’t know, maybe it isn’t water, I don’t even know what it might be. It doesn’t give you much confidence.

- I think it’s… sausages and all that. Since I started reading the labels, I haven’t bought them again. I haven’t bought them again because it makes me uneasy. But packaged meat, well, sometimes, yes”.

*(Tíjola focus group)*

Processed products that are considered highly artificial or which contain large amounts of chemical additives—such as industrial pastries, crisps, packaged juices and soft drinks—provoke great suspicion. This is especially the case with industrial pastries, due on the one hand to the presence of saturated fats and sugars and, on the other, to the existence of chemical additives such as sweeteners, colourants, flavourings and preservatives.

“Looking after children, I think, is much more complicated than controlling your own diet. The children who eat worse than... Well, I don’t know if it’s worse, but they eat what is sold as food for children, and it has such an enormous amount of sugar; I always say, go to the supermarket and get a My First Danone, and a normal Danone, and look at the amount of sugar in My First Danone. Your heart sinks. And it is very difficult to escape from those kinds of foods, no matter how much you want to. At home you can give them the healthiest, really the healthiest food, look out for them... But they are in the world… It’s really very difficult”.

*(Antequera focus group)*

In a similar way to the above-mentioned groups, there was an ambivalent associative subdomain in the participants’ (mis)trust in food depending on its origin, manipulation and distribution. Concerning meat, the mothers distrusted meat from animals that had been fed with fodder, with “chemical substances” or that had been injected with hormones or other drugs. Instead, they preferred meat that was more “natural”, from animals allowed to freely graze in the open.

“The fewer the additives, the fewer the extra preservatives that aren’t natural, for me that’s closer to being healthy. I mean, for example, before on the label there was the word ‘tuna’, which is a fish that, within a balanced diet… fish, well, you consider it... well, me at least, I consider it part of a balanced diet. But it’s not a fish that gives me much confidence, because it reminds me of mercury, pollution, all that. And I’d almost rather eat canned tuna or tuna that comes from a fish farm, than the natural fish, because, perhaps because of the context of mercury that’s been related with tuna and suchlike… it depends on the food. The more natural, the less it’s packaged, the fewer preservatives added, the more natural it is. For me, that’s the best”.

*(Antequera focus group)*

There was a tendency to distrust large fish and to prefer smaller ones, caught near the coast, since there is a widespread belief that big fish may have consumed more heavy metals and mercury.

Finally, one area of item grouping in the *nMDS* model of distrust was spatially separated from the rest. This associative subdomain was related to (mis)trust in relation to food preservation. For example, in the narratives obtained from the focus groups, the case of frozen fish highlights the doubts and uncertainty created by this form of food preservation. Although most of the women believed they can trust this food, because freezing the fish keeps it in a good state of preservation, they also feared that there may have been a break in the cold chain before it reached the consumer. The doubts raised by this concern, not knowing whether the frozen food maintains the same properties as fresh fish, makes them somewhat distrust it.

“But when you’re pregnant, you get more cautious. You wash the vegetables much better than before. You even freeze the food. Maybe you’re not pregnant and you say, “Well, look, I eat fresh fish, freshly cooked”. But now, being pregnant, it’s different; I prefer to freeze it. You do that, whatever, just to be on the safe side…”.

*(Antequera focus group)*

## 4. Discussion

Pregnancy and lactation are vital stages in a woman’s life, during which food becomes a central issue in relation to her health, and the changes in her body are experienced with fear and trepidation [17]. These feelings are associated with the fact that mothers, in this cycle of their lives, are marked by the discourse of risk and the precautionary principle as strategies to manage uncertainty [18] and to protect the foetus or the baby [19].

Food insecurity can affect all stages of life. However, women are at greater risk of suffering from this condition due to gender determinants as the result of inequalities arising from risk factors such as gender violence or unequal access to employment and education [47]. A balanced diet and appropriate weight gain during pregnancy are associated with better maternal and perinatal outcomes. Therefore, weight gain and nutrition are significant areas of public health concern during this stage of life [48]. Conversely, overnutrition and undernutrition both increase the risk of an adverse perinatal outcome, including excessive or inadequate foetal growth, gestational diabetes mellitus, preterm birth and pre-eclampsia. Furthermore, the absence of proper nutrition in early childhood can have life-long consequences. Therefore, it is important to explore the social representations around ‘healthy eating’ of these women and the factors that influence their trust or distrust in food.

The pregnant and breastfeeding mothers consulted expressed great concern about the quality of the food they consume and about its possible effects on their own health and on that of their child. Regarding which foods they can trust, these women must sometimes make highly complex decisions. The participants in our study emphasised the importance of reading food labels, of knowing the composition of the products consumed and of seeking information on any foods that might seem suspicious. However, they also recognised that worrying excessively about food quality could mean, ultimately, not eating anything.

In addition, the participants mentioned other factors that make it even more difficult to decide which foods to choose. They realise that caution is more important than the pleasure of eating. Therefore, although they like many of the foods referred to with distrust, for reasons of health they prefer to avoid them. Some women also observed that the amount of food and the frequency of its consumption are important factors, and that any excessive consumption can be harmful.

In the opinion of the pregnant and breastfeeding mothers consulted, an adequate diet is one based on the consumption of fruits and vegetables (preferably fresh) and of organic meats. However, these preferences are not always applied in practice since other factors such as price and availability (not addressed in this study) must also be taken into account. Although the medical–nutritional discourse based on sustainability has steadily gained strength among consumers as a significant criterion in their food choices [49], this environmental discourse (recommending a diet based on the production and consumption of organic meats, fruits and vegetables) does not constitute the subdomain most frequently identified in our analysis. Proximity and taste remain the preferred criteria for choosing fruits and vegetables, while “fresh” is considered synonymous with “natural” and with not having undergone cold storage for preservation.

Legumes and cereals are easier to store and hence are more generally trusted than other foods. In no case did the study participants question the production process for cereals (with the exception of transgenics) or legumes. On the contrary, although eggs and dairy products are trusted foods, they are often viewed with some suspicion due to industry involvement in the manufacturing process.

Fish and meat generate serious concern as their properties are considered ambivalent depending on the food’s origin and mode of production. According to the UN Food and Agriculture Organisation [50], Spain is the fourth country in the world in fish consumption, with an annual per capita consumption of 45.6 kg. The origin and type of fish products—from various countries, fresh or frozen, large and small—creates some disquiet. Medical discourses favour the consumption of small fish in order to enhance the nutritional properties of this type of food [51] and advise against the consumption of large fish, which are subject to the risk of mercury contamination.

Ambivalence among the participants regarding the consumption of meat is due to the high consumption of sausage products in Spain. Many types are specifically warned against during pregnancy. Ambivalence is also due to the influence of discourses in the media concerning how the animals are fed [52] and because of medical warnings about effects on cardiovascular health [53].

In summary, the women consulted in this study expressed ambivalence between trust and distrust in meat, fish, dairy products and eggs, mainly regarding the ways in which these foods are produced. The participants, however, were in close agreement in classifying prepared or precooked products, basic processed products and highly artificial products with added chemical substances as foods to be distrusted due to suspicions about the manufacturing process.

## 5. Conclusions

The pregnant and breastfeeding women in our study groups classified various foods and assigned certain attributes to them according to the level of trust and mistrust inspired in each case, thus providing a social representation of food risks. These criteria are perceived by women as relevant to their food decisions and, therefore, emic knowledge should be taken into account when developing food safety programmes and planning actions aimed at pregnant and breastfeeding women.

We believe that achieving a better understanding of what women consider ‘healthy eating’ and determining the factors that influence their trust or distrust in the food consumed is critical. This is one of the main contributions of this article. These findings could be very useful for health professionals, revealing which criteria determine trust/mistrust among this population group and highlighting similarities and differences with regard to medical–nutritional recommendations.

## Figures and Tables

**Table 1:** First phase. Sociodemographic characteristics of the participants.

		Pregnant Women	Breastfeeding Women
Age	20–29 years	3	2
	30–39 years	33	23
	40 years and over	4	6
Number of children	1	18	15
	2	18	12
	3 or more	4	4
Education level	Primary	3	0
	Secondary	11	8
	Higher	26	23
Place of residence	Catalonia	29	22
	Andalusia	11	9

**Table 2:** Second phase. Sociodemographic characteristics of the participants.

		Pregnant Women	Breastfeeding Women
Age	20–29 years	5	5
	30–39 years	22	27
	40 years and over	2	1
Number of children	1	16	28
	2	10	4
	3 or more	3	1
Education level	Primary	2	2
	Secondary	9	10
	Higher	18	21
Place of residence	Catalonia	7	19
	Andalusia	22	14

**Table 3:** Food categories associated with trust (*n* = 20) and distrust (*n* = 20) in the pile sorts obtained from the free listings.

Trust	Distrust
Fruit Vegetables Legumes Fish Meat Cereals Meal Nuts Bread Pasta Dairy products Eggs Rice Chicken Yoghurt Fresh fish Organic meat Organic products Fresh vegetables Water	Industrial pastries Pre-cooked food Sausage products Frozen food Crisps Ready-made sauce Frankfurters Fast food Packaged meat Packaged juice Packaged food Sweets Processed meat Soft drinks Tuna Meat Fish Canned food Supermarket meat Ready-to-eat meals

**Table 4:** Clusters or dimensions in the *nMDS* related to trust (*n* = 5) and distrust (*n* = 5) in food items.

Trust	Distrust
Items inspiring confidence due to the nutritional properties of the food itself and/or its origin and manipulation. Items characterised by ambivalence regarding trust/distrust. Cereals, legumes and nuts. Eggs Dairy products	Prepared or precooked products. Processed basic foods. Highly artificial processed products with many added chemicals. Items that are distrusted due to their origin, manipulation and/or distribution. Items that are distrusted due to worries about food preservation.

## Data Availability

The data presented in this study are not publicly available due to privacy and confidentiality reasons.
